# Clinical challenges in the management of isolated GH deficiency type IA in adulthood

**DOI:** 10.1530/EDM-13-0057

**Published:** 2014-02-01

**Authors:** Anna Casteràs, Jürgen Kratzsch, Ángel Ferrández, Carles Zafón, Antonio Carrascosa, Jordi Mesa

**Affiliations:** Department of EndocrinologyHospital Universitari Vall d'Hebron, Universitat Autònoma de BarcelonaPg. Vall d'Hebron 119-129, Barcelona, 08035Spain; 1 Institute of Laboratory Medicine, Clinical Chemistry and Molecular Diagnostics, University of LeipzigLeipzigGermany; 2 Department of PediatricsAndrea Prader Centre, Hospital Universitario Miguel ServetZaragozaSpain; 3 Department of PediatricsHospital Universitari Vall d'Hebron, Universitat Autònoma de BarcelonaBarcelonaSpain

## Abstract

Isolated GH deficiency type IA (IGHDIA) is an infrequent cause of severe congenital GHD, often managed by pediatric endocrinologists, and hence few cases in adulthood have been reported. Herein, we describe the clinical status of a 56-year-old male with IGHDIA due to a 6.7 kb deletion in *GH1* gene that encodes GH, located on chromosome 17. We also describe phenotypic and biochemical parameters, as well as characterization of anti-GH antibodies after a new attempt made to treat with GH. The height of the adult patient was 123 cm. He presented with type 2 diabetes mellitus, dyslipidemia, osteoporosis, and low physical and psychological performance, compatible with GHD symptomatology. Anti-GH antibodies in high titers and with binding activity (>101 IU/ml) were found 50 years after exposure to exogenous GH, and their levels increased significantly (>200 U/ml) after a 3-month course of 0.2 mg/day recombinant human GH (rhGH) treatment. Higher doses of rhGH (1 mg daily) did not overcome the blockade, and no change in undetectable IGF1 levels was observed (<25 ng/ml). IGHDIA patients need lifelong medical surveillance, focusing mainly on metabolic disturbances, bone status, cardiovascular disease, and psychological support. Multifactorial conventional therapy focusing on each issue is recommended, as anti-GH antibodies may inactivate specific treatment with exogenous GH. After consideration of potential adverse effects, rhIGF1 treatment, even theoretically indicated, has not been considered in our patient yet.

**Learning points:**

- Severe isolated GHD may be caused by mutations in *GH1* gene, mainly a 6.7 kb deletion.
- Appearance of neutralizing anti-GH antibodies upon recombinant GH treatment is a characteristic feature of IGHDIA.
- Recombinant human IGF1 treatment has been tested in children with IGHDIA with variable results in height and secondary adverse effects, but any occurrence in adult patients has not been reported yet.
- Metabolic disturbances (diabetes and hyperlipidemia) and osteoporosis should be monitored and properly treated to minimize cardiovascular disease and fracture risk.
- Cerebral magnetic resonance imaging should be repeated in adulthood to detect morphological abnormalities that may have developed with time, as well as pituitary hormones periodically assessed.

## Background

Isolated growth hormone deficiency (IGHD)-associated short stature is estimated to occur in 1 in 4000–10 000 individuals (1). Although most cases are considered to be sporadic, a genetic cause is found in about 10–15% of the patients presenting with marked short stature (height SDS <−4.5) or having an affected first-degree relative (up to 40% of the familial cases) (2). Genetic testing is also recommended in cases exhibiting normal pituitary morphology on magnetic resonance imaging (MRI). So far, known genetic factors responsible for IGHD include defects in *GH1* gene or GH-releasing hormone receptor (*GHRHR*) gene, more rarely mutations in Bruton's tyrosine kinase (*BTK*) gene, or mild phenotypes of mutations in *HESX1* or *SOX3* gene. Besides other pituitary hormone deficiencies, mutations in *PROP1* and *POUF1* genes should also be considered in childhood. Inheritance patterns and GH disturbance severity have helped traditionally to classify IGHD into four clinical types (IA, IB, II, and III); however, the molecular heterogeneity within each subtype makes this classification cumbersome (3) (4) (Table 1).

**Table 1:** Classification of isolated GH deficiency

**IGHD type**	**Genetic abnormalities**	**Inheritance**	**GH levels**	**Growth response to GH**	**Pituitary imaging**	**Comments**
IA	*GH1* gene deletions and nonsense mutations	AR	Absent	Transitory	AP: normal or hypoplastic	Often have anti-GH antibodies after GH replacement
					PP: eutopic	
IB	*GH1* gene splice site mutations	AR	↓	Yes	AP: normal or hypoplastic	
	*GHRHR* gene mutations				PP: eutopic	
II	*GH1* gene splice site and missense mutations and intronic deletions	AD	↓	Yes	AP: normal or hypoplastic	Variability in height
					PP: eutopic	May develop additional pituitary hormone deficiencies
III	*BTK* or unknown	X-linked	↓	Yes	PP: ectopic or normal	Agammaglobulinemia
						May have mental retardation

AR, autosomal recessive; AD, autosomal dominant; AP, anterior pituitary; PP, posterior pituitary; GHRHR, growth hormone-releasing hormone receptor; BTK, Bruton's tyrosine kinase; ↓, low but detectable.

IGHD type IA (IGHDIA) is characterized by a complete lack of GH production and exhibits the most severe phenotype. The syndrome was first described in 1970 in a group of six patients including a Spanish boy who is the main subject of the case report described herein (5). Underlying genetics are predominantly *GH1* gene deletions, although nonsense or frameshift mutations severely disturbing the GH molecule have also been reported (3). *GH1* gene resides on the 17q22–24 chromosome within a cluster of 65 kb including five homologous genes that predisposes to deletions after unequal recombination and crossing over at meiosis. Appearance of neutralizing anti-GH antibodies (anti-GH Abs) upon recombinant human GH (rhGH) treatment is a characteristic feature of GHDIA (1) (5). As an alternative approach, recombinant human insulin-like growth factor 1 (rhIGF1) is being used in children to improve growth parameters; adult cases have not been reported. Although targeting growth is not an issue in the surveillance of adults with GHD, questions remain with respect to the metabolic disturbances, cardiovascular risk, and lastly longevity.

## Case presentation

A 56-year-old male known to have IGHDIA was referred to our adult endocrinology unit after several years of lapsed follow-up. He was diagnosed in early childhood with severe growth retardation (−5.7 s.d. at 2 years of age). His case was included in pediatric publications as he belonged to the first group of children having the syndrome when it was first described (5). He was born from nonconsanguineous progenitors and any other family member was not of short stature. Birth length at 42 weeks of gestation was already poor (48 cm) and weight was 4080 g. In infancy, he was of proportionate short stature with certain phenotypic features such as puppet-face appearance. We are not aware of hypoglycemic episodes or micropenis in childhood and he had no problems related to dentition or puberty. Other pituitary hormonal axis functions were normal, and initial pituitary image did not reveal major abnormalities. Genetic study revealed a severe *GH1* gene deletion of 6.7 kb in homozygous state. At the age of 7 years, with a −6 s.d. growth delay, he was given GH extracted from human pituitaries based on the Raben method in Zürich Children Hospital (Switzerland) for a couple of years, but no significant increase in growth was achieved (−6.2 s.d. at 9 years of age), compatible with the development of anti-GH Abs. Thus, his final height was poor, reaching only 123 cm, representing a SDS of −8.46 with respect to the Spanish population. Of interest is the fact that he leads a normal life, runs a bar, is married, and has two sons (carriers of 6.7 kb deletions without any clinical expression). He has been a heavy smoker for the last 30 years. Now in his fifties, he presents with health complaints that resembles those of adult GHD. He suffers from asthenia, osteomuscular pain, and osteoporosis.

**Figure 1:**
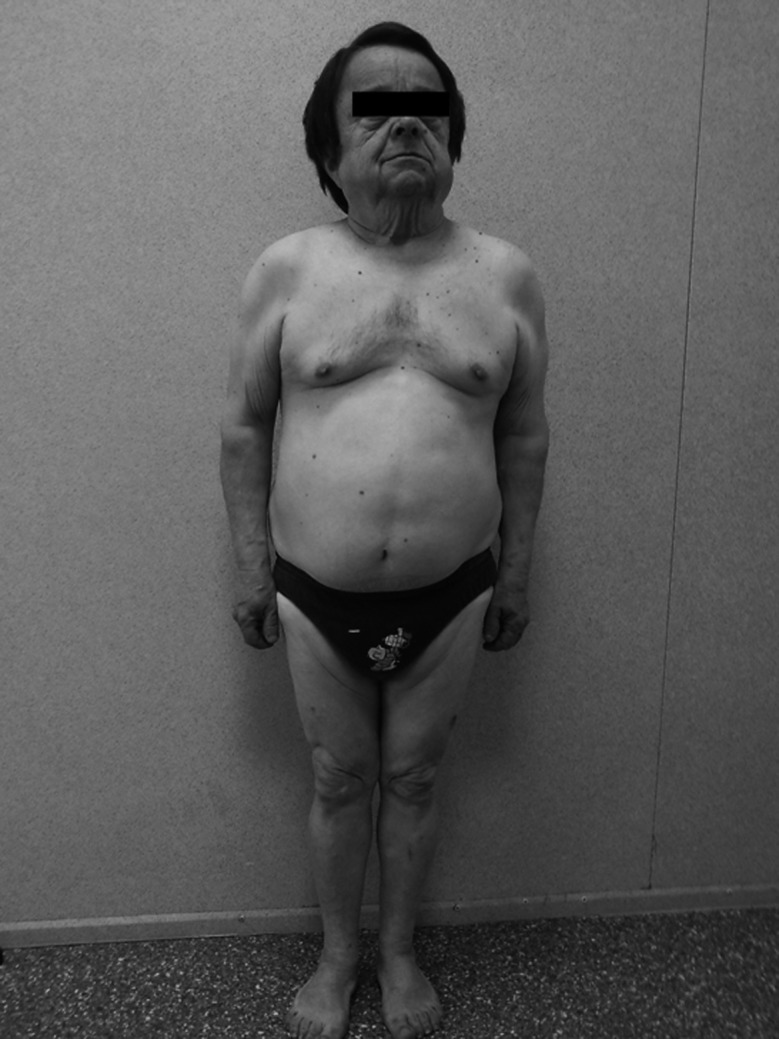
Fifty-six-year-old male patient, with 6.7 kb deletion in *GH1* gene and height 123 cm.

On physical examination (Fig. 1), apart from severe short stature, he was found to weigh 38 kg (BMI 24.71), with waist circumference being 85.5 cm, hip circumference 83 cm, and blood pressure 137/71 mmHg. He did not have any sign of hypogonadism. Genitals were normal with a testicular size of 20 ml bilaterally. He had a high-pitched voice.

## Investigation

**Figure 2:**
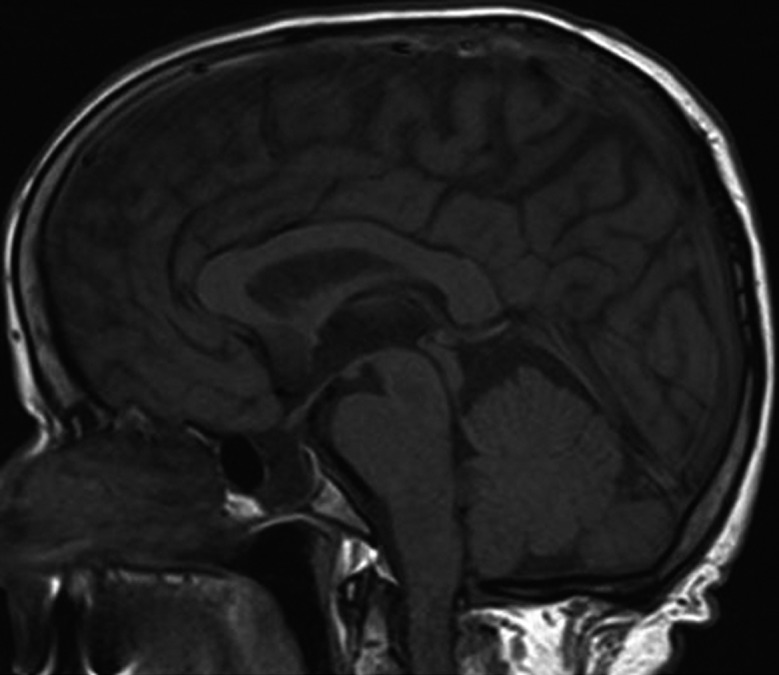
Cerebral MRI, T1-weighted, sagittal midline. Platybasia, short clivus, and small posterior cranial fossa. Arachnoidocele of the sella turcica.

Blood test revealed normal full blood count as well as hepatic and renal function. Diabetes was diagnosed based on a glycohemoglobin level of 6.8% with basal glucose levels of 128 mg/dl; hypercholesterolemia was also present: total cholesterol 247 mg/dl (<220), cLDL 182 mg/dl (<130), cHDL 32 mg/dl (>40), and triglycerides 166 mg/dl (43–200). Vitamin D deficiency with secondary parathyroid hormone (PTH) elevation and normal calcemia was observed: 25OH vitamin D3 6.7 ng/ml (6.6–46.4), PTH 140 pg/ml (7–82), calcium 9 mg/ml, and phosphorus 2.8 mg/dl. The following hormones were present within the normal range: thyroid-stimulating hormone 2.49 mU/l (0.4–4), free thyroxine 1.05 ng/ml (0.7–1.6), luteinizing hormone 2.6 U/l (0.8–8), follicle-stimulating hormone 3.6 U/l (0.7–12), total testosterone 484 ng/dl (241–827), prolactin 3.9 ng/ml (2.1–17.7), cortisol 14 μg/dl (4.3–22.4), and adrenocorticotropic hormone 12 pg/ml (5–46). The presence of a clearly suppressed GH axis was remarkable: GH <0.05 ng/ml (<8 ng/ml), IGF1 <25 ng/ml (69–252), and IGF-binding protein 3 (IGFBP3) <0.5 mg/l (3.1–7.9). On cardiovascular evaluation, carotid intima media thickness was found to be within the normal range, 0.6 mm in both carotids. Atherosclerotic plaques without hemodynamic repercussion were detected in both carotid bulbs. Further evaluation of biochemical cardiovascular risk parameters revealed the following: HOMA-IR 2.2 (insulin 6.5 mU/l (3–25) and glucose 146 mg/dl), HOMA-β 28.3%, C-reactive protein 0.78 mg/dl (<50), and lipoprotein (a) 2.9 mg/dl (<20). Bone densitometry revealed osteoporosis (*T*-score −2.7 on lumbar spine and −3.1 on femoral neck). A score of 18 points was obtained for quality of life assessed with the QoL–AGHDA questionnaire. New pituitary MRI done (Fig. 2) showed flattening of the skull base in relation to platybasia and also a short clivus and a small posterior cranial fossa, but without herniation of the cerebellar tonsils below the foramen magnum. There was an arachnoidocele of the sella turcica enlarging the bony structure and displacing the pituitary gland toward the posterior margin.

## Treatment

As the patient was treated with a quite impure immunogenic GH preparation rather than with the current recombinant GH hormone formulations in childhood, we tried to treat him again with adult GH dosing, 0.2 mg daily (Genotonorm; Pfizer, Inc., New York, NY, USA). After 1 month of treatment, there was no increase in IGF1 or IGFBP3 levels. Then, an empirical IGF1 generation test was carried out at higher doses (0.03 mg/kg per day=1 mg GH/day for 5 days), without achieving any further response (GH 33.3 ng/ml and IGF1 <25 ng/ml). Antibodies against GH were determined before and 3 months after treatment using a modified radioprecipitation assay. The antibodies exhibited detectable binding activity even basally, i.e. after 50 years of exposure, and their levels increased tremendously with GH replacement. Anti-GH Ab levels were initially 101 U/ml (titer 1:10; reference range <6.03 U/ml) and increased to >200 U/ml (titer 1:10 000). Owing to the lack of biochemical response or clear clinical benefit, GH therapy was discontinued. Thus, comorbidities were treated appropriately with dietary education, metformin, aspirin, statin, and calcium plus vitamin D3 supplements. The patient refused antiresorptive bisphosphonate treatment for the time being.

## Outcome and follow-up

After 1 year of follow-up, T2DM was under control (latest glycohemoglobin 6.6%), lipid levels were within the target range (cLDL 98), and bone mineral density (BMD) improved 7.3% in lumbar spine and 1.9% in femoral neck.

## Discussion

The patient described herein is, to our knowledge, the oldest adult case of IGHDIA properly described in the literature. Genetic analysis revealed a 6.7 kb deletion in *GH1* gene that entails an absolute lack of GH. Deletion of 6.7 kb has been reported to be the most common mutation accounting for the type IA phenotype (78%); other deletions include 7.6 and 45 kb (3). Less severe mutations in *GH1* gene (splice site and missense) exhibit phenotype IB or II, in which low but detectable GH concentrations are observed and there is response to GH replacement. Type IB can also be caused by recessive *GHRHR* mutations. Type IA is less frequent than type IB or type II, accounting for only about 5% of the cases (4). However, in the work carried out by Wagner *et al.* (2), the absolute frequency of *GH1* gene deletions in a cohort of severe short stature (<4.5 s.d.) was 12.5% (19/151), especially in those of an Asian origin.

Phenotypic appearance of GHDIA includes an already intrauterine growth retardation (birth length <49 cm, considerably shorter than nonaffected siblings) and severe growth delay in childhood, even beyond −4.5 SDS. Adult height is clearly small, but quite variable in the few adult cases reported in the literature (130–140 cm), as some do not produce anti-GH Abs or would not have received rhIGF1 therapy in childhood (6) (7). Cherubic facial features derive from midfacial hypoplasia and forehead prominence. Small posterior fossa and platybasia described in our patient's MRI may also reveal bone effects of lifelong GHD. On the other hand, the pituitary arachnoidocele found in the MRI is meant to have developed with time; possibly, it is merely a coincidental finding, but more information about adult IGHDIA cases is lacking. Generally, cases with mutations in *GH1* gene have normal pituitary morphology in contrast to cases with other causes of congenital GHD such as perinatal insults that are associated with alterations in the pituitary stalk or ectopic posterior pituitary lobe. However, some cases present with anterior hypoplasia, and it is not ruled out that pituitary image findings may evolve with time. Oliveira *et al*. (8) reported anterior pituitary hypoplasia in patients with inactivating *GHRHR* mutations, compatible with the lack of GHRH effect on somatotrophs. Our patient has not developed any other hormonal deficiency until now. Development of additional pituitary hormone deficiencies in type II IGHD has been attributed to the disrupting effects of the aberrant predominance of 17.5 kDa GH isoform on the pituitary gland (9) and thus this would not be the case in type IA.

The absence of endogenous production of GH in IGHDIA explains the immune response upon rhGH administration; however, the appearance of antibodies may not be a regular finding even among members of the same family. Moreover, anti-GH Abs may have different binding affinities, compromising GH coupling to the receptor. *GH1* mutation severity, HLA haplotypes, or other immune response genes, as well as age at first antigen contact, could contribute to these differences in immune tolerance. There have been cases of anti-GH Abs disappearing and years thereafter allowing new response to GH treatment (6). Interestingly, in the present case, the antibodies had persisted at a low level for many years and their levels had increased considerably after a new contact with GH. Efforts to remove anti-GH Abs with plasmapheresis or treatment with IgG or cyclophosphamide have been frustrating and nor has desensitization using small amounts of exogenous GH prevented their appearance (6).

Physiologically, GH exerts its action on the peripheral tissues both directly and indirectly with the mediation of circulating and locally produced IGF1. Severe IGHDIA with extremely low levels of circulating IGF1 could exhibit reasonable similarities with Laron's dwarfism, where a mutation in GH receptor abolishes the effect of GH on IGF1 production. Recombinant IGF1 (rhIGF1) represents an interesting treatment option for children with IGHD1A developing antibodies or GH insensitivity syndrome (7). rhIGF1 is not licensed for use in the treatment of adult GHD; however, in view of the very severe ‘secondary IGF1 deficiency’, it seems plausible to consider treating our patient with rhIGF1. Reports of children with type IA is quite recent and scarce (6) (10) and sometimes disappointing, with them not achieving a proper catch-up in growth in some cases or exhibiting secondary adverse effects on lipids and BMI.

It is well known that GHD symptoms in adulthood include changes in body composition with an increase in fat body mass in detriment of lean one, decreased bone mass, unfavorable lipid profile, reduced physical and cardiac performance, premature atherosclerosis, and poor quality of life.

BMD in short individuals measured by the standard two-dimensional method may yield false results, oversizing osteoporosis. Bone mineral apparent density (volumetric density) allows the calculation of true density, as a bone mass/volume (11). Thus, we calculated vertebral (L2–L4) volumetric density as bone mineral content per bone volume (area of vertebrae (cm^2^)×height). Volumetric density was 0.35 g/cm^3^, compatible with a much better bone status than obtained by areal density, although a *Z*-score for his age and gender group is currently not available.

While GHD or insensitivity might be associated with hypoglycemia in children, in adults hyperglycemia is common. The age-related decline in insulin sensitivity in GH-deficient patients is not well understood yet; however, we chose to treat the patient with metformin for T2DM, as it was the most appropriate drug. Insulin resistance may be the main underlying mechanism for diabetes in our patient, after central adiposity, older age, and HOMA-IR value. Nevertheless, recent studies in Laron's dwarfs and in patients with *GHRHR* gene mutation have reported normal or reduced HOMA-IR values and elevated adiponectin levels, despite concomitant obesity (12) (13). Insulin secretion seems to be also compromised in the latter group after low HOMA-β levels (mean 28.1%), a value similar to that observed in the present case. It is hypothesized that significantly reduced IGF1 levels could compromise β-cell mass. In summary, IGHD would not protect from the development of diabetes as postulated.

Information on other cardiovascular risk factors in IGHD is scarce, but no premature atherosclerosis signs have been found in untreated type IB patients due to *GHRHR* mutations in a Brazilian kindred (14). On the contrary, the patient described herein had carotid atherosclerotic plaques, indicating slow progression of atherosclerosis. This finding may well have a multifactorial origin.

Cancer reports are also anecdotic among Laron's and IGHD patients. Reduced IGF1 signaling has been reported to have a protective effect on aging in some animal models. However, this cannot be extrapolated to humans, due to a much more complex endocrine/paracrine network being involved in organ and tissue development as well as energy homeostasis throughout life. It is fairly accepted that in the post-developmental stage of life, GH and IGF1 have numerous beneficial actions in skeletal muscle and cardiovascular and nervous systems, but a negative effect on insulin sensitivity and cancer risk. There is less information on the longevity of GHDIA-affected patients, except for a report by Besson *et al*. (15). Life span was found to be greatly reduced in 11 IGHDIA individuals compared with nonaffected siblings and normal population of the same time and place (Switzerland, late 19th–early 20th century). Reported survival in GHDIA patients vs controls was 56 vs 75 years for men and 46 vs 80 years for women. The causes of death did not vary between groups: cardiovascular and infections. Of note, there were no records on cancer or diabetes, although information sources were not medical. In contrast to acquired hypopituitarism cases, congenital severe GHD cases have much lower serum IGF1 levels and have never been exposed to GH and thus they may not be equivalent in terms of GH/IGF1 axis disturbances and health prognosis.

In conclusion, establishing the etiologic diagnosis of GHD in childhood is a major challenge requiring a combination of clinical, auxological, biochemical, and pituitary imaging data. Genetic testing is recommended mainly in case of severe growth delay, familial affected members, or normal MRI, but exceptions to the rule have to be considered. This case of IGHDIA shows the persistence of anti-GH Abs 50 years after the exposure to GH and the long-lived immune intolerance despite treatment with recombinant GH preparations. Adult GH absence and the consequent severe IGF1 deficiency may underlie the patient's present metabolic disorders, osteomuscular symptoms, and reduced quality of life. However, at the moment, we choose not to treat the patient with rhIGF on the basis of risk–benefit.

IGHDIA is an exceptional *in vivo* model to study GH/IGF1 actions throughout life. Limitations to extending conclusions in terms of diabetes, cancer, and longevity are the few reported cases due to the rarity of the disease and the multiple *in vivo* biases such as particularities within endogamic kindreds or certain life styles.

## Patient's perspective

Patient account/experience.

## Patient consent

Written informed consent was obtained from the patient for publication of this case report and any accompanying images.

## Author contribution statement

Anna Casteràs, MD, supervised and treated the patient. She searched pre-existing literature, wrote the case report and the discussion, and edited the final version of the paper. Jürgen Kratzsch, MD, PhD, carried out the analysis of the anti-GH antibodies and participated in the critical revision of the paper. Ángel Ferrández, MD, PhD, diagnosed and treated the patient in childhood and contributed to the critical revision of the case report. Carles Zafón, MD, A Carrascosa, MD, PhD, and Jordi Mesa. MD, PhD, participated in the critical revision of the paper.
